# Cell fiber-based three-dimensional culture system for highly efficient expansion of human induced pluripotent stem cells

**DOI:** 10.1038/s41598-017-03246-2

**Published:** 2017-06-06

**Authors:** Kazuhiro Ikeda, Shogo Nagata, Teru Okitsu, Shoji Takeuchi

**Affiliations:** 1 Institute of Industrial Science, The University of Tokyo, Tokyo, Japan; 2 ERATO Takeuchi Biohybrid Innovation Project, Japan Science and Technology Agency, Tokyo, Japan; 3 Graduate School of Life and Environmental Sciences, University of Tsukuba, Ibaraki, Japan

## Abstract

Human pluripotent stem cells are a potentially powerful cellular resource for application in regenerative medicine. Because such applications require large numbers of human pluripotent stem cell-derived cells, a scalable culture system of human pluripotent stem cell needs to be developed. Several suspension culture systems for human pluripotent stem cell expansion exist; however, it is difficult to control the thickness of cell aggregations in these systems, leading to increased cell death likely caused by limited diffusion of gases and nutrients into the aggregations. Here, we describe a scalable culture system using the cell fiber technology for the expansion of human induced pluripotent stem (iPS) cells. The cells were encapsulated and cultured within the core region of core-shell hydrogel microfibers, resulting in the formation of rod-shaped or fiber-shaped cell aggregations with sustained thickness and high viability. By encapsulating the cells with type I collagen, we demonstrated a long-term culture of the cells by serial passaging at a high expansion rate (14-fold in four days) while retaining its pluripotency. Therefore, our culture system could be used for large-scale expansion of human pluripotent stem cells for use in regenerative medicine.

## Introduction

Human pluripotent stem cells, including embryonic stem (ES) cells^1^ and induced pluripotent stem (iPS) cells^2^, are capable of expanding indefinitely and differentiating into cells from all three germ layers. Thus, they are considered to be a useful cell source for application in the field of regenerative medicine^3^. In practical use, human pluripotent stem cell-derived cells are often required in large numbers^4–6^. For example, in cell transplantation, approximately 10^9^ cardiomyocytes are required for treating myocardial infarction, approximately 10^9^ insulin-producing β cells for type 1 diabetes mellitus, and approximately 10^10^ hepatocytes for hepatic failure^4^. To obtain these large numbers of cells, the development of scalable culture systems with efficient expansion of human pluripotent stem cells is needed before subsequent differentiation steps^6^.

Suspension culture system is well-known as a scalable method and is considered to be applicable for large-scale cell culture of human pluripotent stem cells^4, 6–14^. In this system, the cells can proliferate and spontaneously form spherical aggregations that provide a three-dimensional (3D) microenvironment for the cells. However, controlling the thickness of the cell aggregations is difficult due to the fusion of aggregates and/or cellular proliferation; increase in the thickness may cause limited diffusion of gases and nutrients into the aggregations, resulting in cell death and low expansion rate^4, 8, 14, 15^.

**Figure 1:**
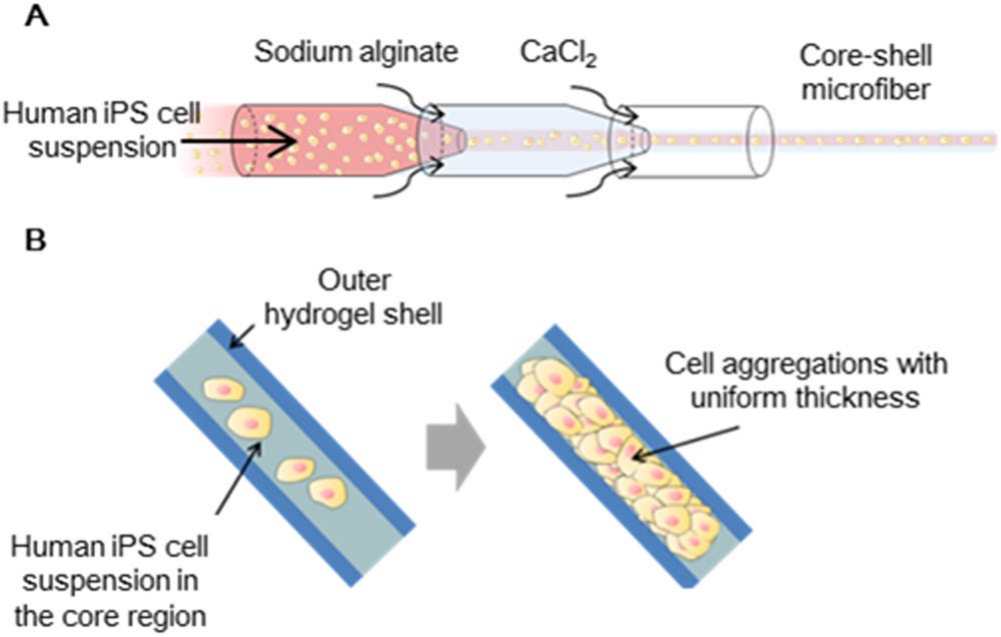
Schematic illustration of the core-shell microfiber culture system for human induced pluripotent stem (iPS) cells. (**A**) An illustration of the double co-axial laminar flow microfluidic device used for the formation of the human iPS cell-laden core-shell hydrogel microfiber. (**B**) The cells encapsulated within the core-shell microfibers proliferate and form cell aggregations with uniform thickness by expanding along the microfiber.

In this study, we developed a method to restrict the increase in the thickness of human iPS cell aggregations during cell expansion using the cell fiber technology. This technology enables encapsulation of the cells suspended in the culture medium or extracellular matrix (ECM) solution into the core-shell hydrogel microfibers^16, 17^ (Fig. 1A). In the core of the microfiber, the cells can form aggregates and expand along the microfiber, and the thickness of the aggregations is constrained by the hydrogel shell (Fig. 1B). This method allows sufficient exposure through the semipermeable shell of encapsulated cells to gases and nutrients^18^, leading to efficient expansion with high viability. Here, we first investigated cell viability in the cell fiber-based culture system (termed “core-shell microfiber culture system”). We then optimized the core components, including the type of ECM and the initial cell density within the core region of the microfibers, to promote expansion rates of the cells with pluripotency. Finally, we demonstrated long-term cell proliferation by serial passaging while sustaining a high expansion rate and pluripotency using our culture system.

## Results and Discussion

### Comparison between the core-shell microfiber culture system and the suspension culture system of human iPS cells

**Figure 2:**
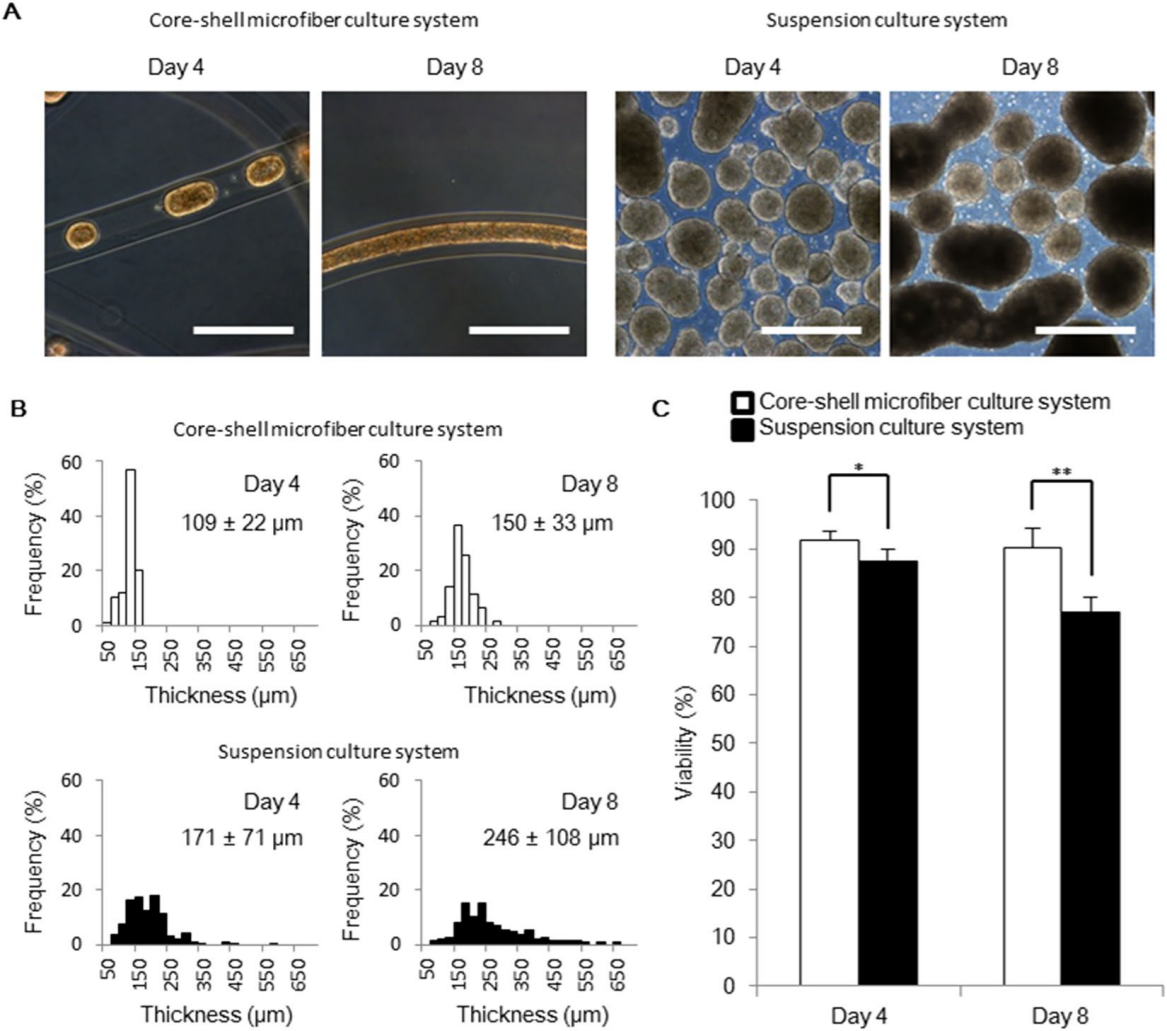
The thickness and the viability of human iPS cell aggregations in the core-shell microfiber culture system. (**A**) Microscopic images showing the morphologies of human iPS cell aggregations in the core-shell microfiber culture and the suspension culture systems on day 4 and 8. Scale bar: 500 μm. (**B**) Thickness distributions of cell aggregations in the core-shell microfiber culture and the suspension culture systems on days 4 and 8. The thickness was calculated by measuring the minor axis of cell aggregation regarded as an ellipse. (**C**) The cell viabilities in the core-shell microfiber culture and the suspension culture systems after the retrieval of cell aggregations from the microfibers and dissociation of cell aggregations into single cells on days 4 and 8 (N ≥ 3). *P < 0.05.

To demonstrate the function of the core-shell microfiber culture system that can restrict the increase in the thickness of cell aggregation and can subsequently maintain cell viability, we compared this system with the conventional suspension culture system. We used core-shell microfibers encapsulating human iPS cells that were suspended in the culture medium without any additional ECM components to adjust the culture condition to that of the conventional suspension culture system, where no ECM components are used^12^. Moreover, the initial cell density in the core of the microfibers was fixed to 1.0 × 10^7^ cells/mL, and the same number of cells and same amount of medium were used. As a result, the aggregation thicknesses were 109 ± 22 μm in the core-shell microfiber culture system and 171 ± 71 μm in the suspension culture system on day 4, and the aggregation thicknesses were 150 ± 33 μm in the core-shell microfiber culture system and 246 ± 108 μm in the suspension culture system on day 8 (Fig. 2A and B), showing that our core-shell microfiber culture system restricted the increase in the thickness of the cell aggregations. Moreover, cell viability was significantly higher in the core-shell microfiber culture system than that in the suspension culture system, both on day 4 and day 8 (Fig. 2C); we observed that non-viable cells in the suspension culture system were mostly located in the central region of the aggregations (Supplementary Fig. S1). The high cell viability in our culture system could be attributed to the restricted thickness of the aggregation that allows cells to be exposed to sufficient gases and nutrients^14, 19, 20^.

### Optimization of the initial cell density and ECM components in the core regions of the core-shell microfibers to culture human iPS cells

**Figure 3:**
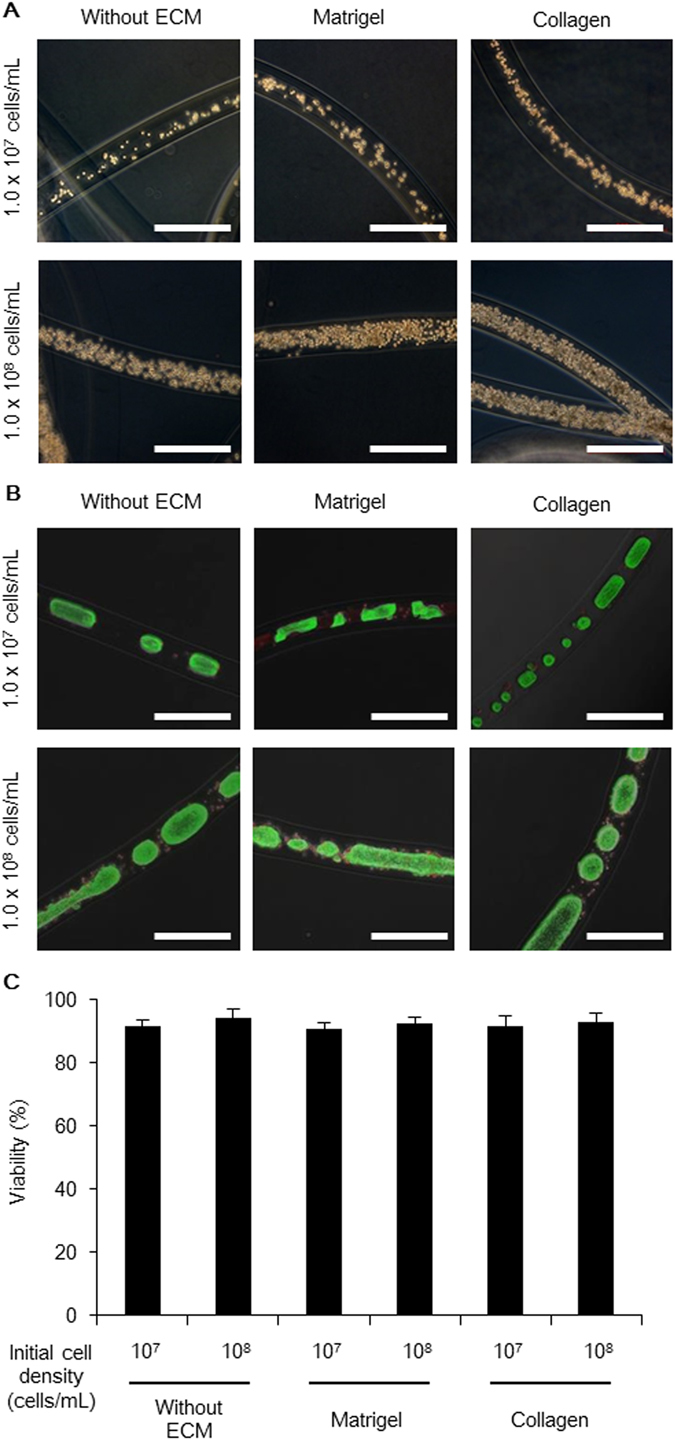
Human iPS cells cultured in various types of core-shell microfibers. (**A**) Phase-contrast images showing the different types of human iPS cell-laden core-shell microfibers immediately after fabrication (day 0). Scale bar: 500 μm. (**B**) Merged phase-contrast and fluorescence images of the six types of core-shell microfibers cultured by day 4. Live cells were stained with calcein AM (green) and dead cells were stained with ethidium homodimer-1 (red). Scale bar: 500 μm. (**C**) Viability of the cells cultured in the six types of microfibers after the retrieval of cell aggregations from the microfibers and dissociation into single cells on day 4 (N ≥ 3).

**Figure 4:**
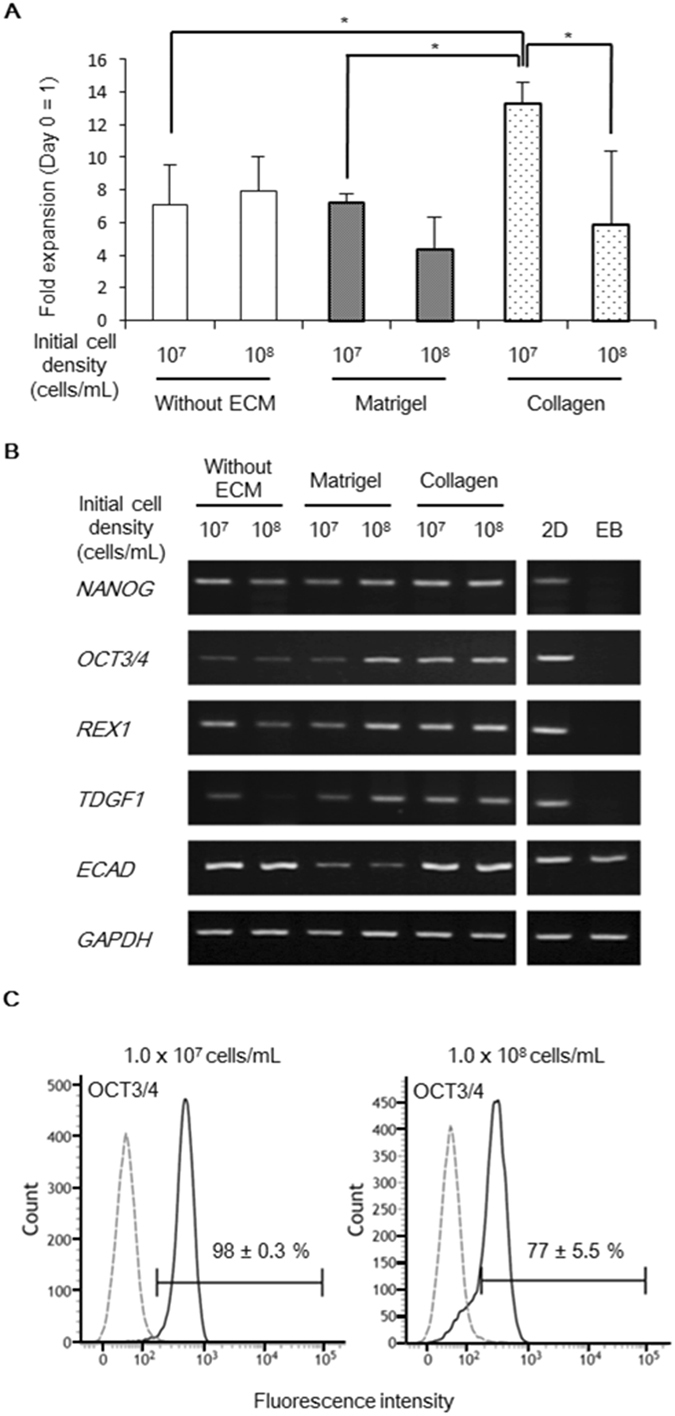
Optimization of the initial cell densities and the extracellular matrix (ECM) components in the core-shell microfibers. (**A**) Expansion rates of human iPS cells in the core-shell microfiber culture systems. The cells were retrieved on day 4, and their expansion rates were calculated (N ≥ 3). *P < 0.05. (**B**) Reverse transcription polymerase chain reaction (RT-PCR) analysis of the pluripotency-associated marker genes expression in cells cultured in six types of human iPS cell-laden core-shell microfibers for 4 days. 2D, cells cultured on a Matrigel-coated culture plate (positive control) and EB, embryoid bodies cultured in differentiation medium (negative control). Full-length gels are presented in Supplementary Figure S9. (**C**) Flow cytometry analysis of OCT3/4 expression in cells cultured with collagen at initial cell density of 1.0 × 10^7^ or 1.0 × 10^8^ cells/mL for 4 days (N = 3). The black solid line and gray dashed line indicate stained cells and the negative control, respectively.

Cell expansion and pluripotency for culturing human pluripotent stem cells are generally influenced by the culture factors, including the initial cell density and ECM components^14, 15, 21–26^. To optimize the factors for human iPS cell expansion while retaining pluripotency in core-shell microfiber culture system, we fabricated six types of core-shell microfibers; in the core region, the initial cell densities of human iPS cells were maintained at low (1.0 × 10^7^ cells/mL) and high (1.0 × 10^8^ cells/mL) values, which was accompanied with two types of ECM components, Matrigel and type I collagen without any ECM components (Fig. 3A). In the preliminary experiment, microfibers at even lower initial cell density (1.0 × 10^6^ cells/mL) resulted in cell death probably because of poor cell–cell adhesion (Supplementary Fig. S2)^27^. We then evaluated the viability, expansion rates, and expression levels of pluripotency-associated markers of the cells cultured in these microfibers. The cells cultured in the all types of microfibers revealed high cell viabilities that ranged from ca. 91% to 94% (Fig. 3B and C), indicating that the core-shell microfiber culture system allows preservation of the cell viabilities regardless of the initial cell densities and the type of ECM components. We found that the expansion rate of human iPS cells accompanied with type I collagen at low initial cell density was higher than the other types of microfibers, and the value of the rate was 13 ± 1.3-fold in 4 days (Fig. 4A). The lower cell expansion rate at high initial cell density with type I collagen may be due to the inhibition of growth of pluripotent stem cells densely packed in the core of the microfiber^28^. In fact, in our culture system, human iPS cells encapsulated in the microfibers at high initial cell density formed aggregations and occupied most of the core space until day 1 (Supplementary Fig. S3).

To evaluate the expression levels of pluripotency-associated marker genes (*NANOG*, *OCT3/4*, *REX1*, *TDGF1*, and *ECAD* genes), we performed reverse transcription polymerase chain reaction (RT-PCR) analysis for the cells cultured in all six types of microfibers. As a result, the human iPS cells accompanied with type I collagen, both at high and low initial cell densities, showed high expression levels of all these marker genes, whereas the cells cultured in other types of microfibers showed a decrease in the expression levels of some of these marker genes (Fig. 4B).

The flow cytometry analysis showed that 98 ± 0.3% of the human iPS cells accompanied with type I collagen at the low initial cell density were OCT3/4-positive. In contrast, 77 ± 5.5% of the cells accompanied with type I collagen at the high initial cell density were OCT3/4-positive (Fig. 4C).

Based on these results, low initial cell density of the cells and the usage of collagen type I are considered to be optimal culture factors for human iPS cell expansion in the core-shell microfiber culture system. Additionally, efficient cell expansion, with the expression of pluripotency-associated markers in the microfibers, supplemented with collagen are supported by a previous study reporting that pluripotent stem cells cultured in type I collagen-rich microenvironment promote cell expansion rate and express high levels of pluripotency-associated markers^29^. The decreased expression of pluripotency-associated markers in the cells cultured in microfibers with collagen at high initial cell density may be due to the tendency for differentiation in conventional 3D culture system at a high initial cell density^12^. We used the optimal ECM and initial cell density in the core-shell microfiber culture system to expand and maintain human iPS cells in the following experiments.

### Serial passaging using the core-shell microfiber culture system

**Figure 5:**
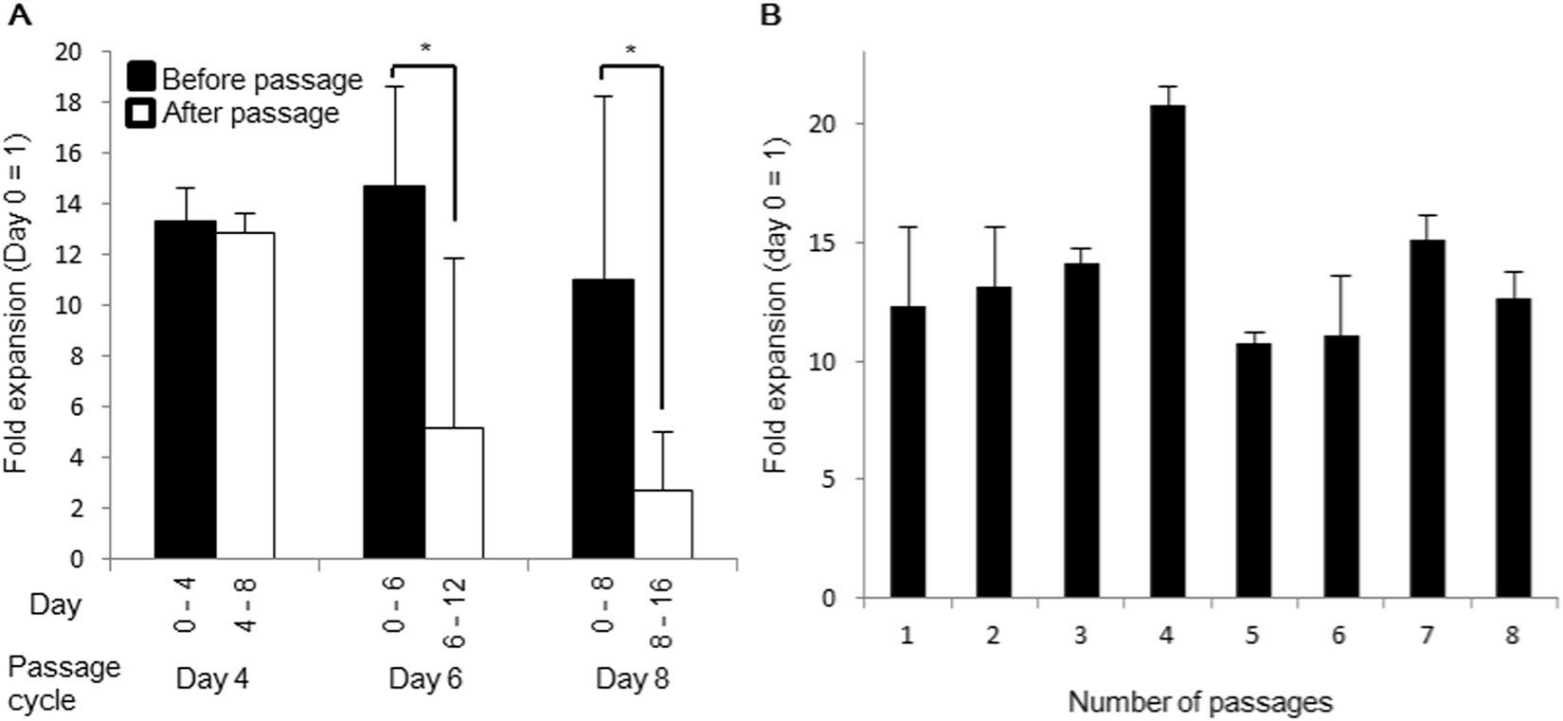
Passaging of human iPS cells in the core-shell microfiber culture system. (**A**) Expansion rates of human iPS cells cultured in the microfibers accompanied with collagen at low initial cell density (1.0 × 10^7^ cells/mL) before and after passage (N ≥ 3). The cells were passaged on day 4, 6, or 8. *P < 0.05. (**B**) Expansion rates of the cells cultured in the microfibers by serial passaging (N ≥ 3). Cells were passaged every 4 days and cultured until 8 passages (32 days).

To achieve highly efficient expansion by serial passaging using the core-shell microfiber culture system, we optimized the passage cycle to maximize the cell expansion rates. As a result, the expansion rates were mostly sustained before and after a passage on day 4 compared with other passage cycles on days 6 and 8 (Fig. 5A), indicating that the passage cycle on day 4 is optimal; human iPS cells reached the growth-arrest on day 8 (Supplementary Fig. S4). Furthermore, the expansion rate of human iPS cells, passaged every 4 days, was maintained at around 14-fold until at least up to eight passages (approximately one month; Fig. 5B). Thus, we estimated the cell expansion rate in our culture system as 1.1 × 10^9^-fold in 32 days, which is, to our knowledge, the highest expansion rate for human pluripotent stem cells in the 3D culture system than previously reported^15^.

The promoted cell expansion in our culture system may reduce the culture period, medium consumption, and cost compared with other scalable culture systems. To achieve the cell quantity required for clinical applications (>1.0 × 10^10^ cells^4, 6^) from 1.0 × 10^6^ cells, the core-shell microfiber culture system will require 16 days and 43 L of medium to reach 3.3 × 10^10^ cells. Moreover, estimated time and quantity of medium required was 24 days and 100 L of medium, respectively, to reach 1.9 × 10^10^ cells in a previously reported suspension culture system^30^. In particular, considering allogeneic transplantation, human pluripotent stem cells and pluripotent stem cell-derived cells must be cultured on an industrial-scale (>1000L)^6^. Our culture system would yield 1.7 × 10^12^ cells with 1000 L of the medium because 6.9 × 10^6^ cells were obtained from 4 mL of the medium, whereas a previously reported suspension culture system yielded approximately 1.0 × 10^12^ cells with the same amount of the medium^30^. Based on these results, we propose that the core-shell microfiber culture system would be suitable for large-scale expansion of human pluripotent stem cells.

### Characterization of human iPS cells in the core-shell microfiber culture system

**Figure 6:**
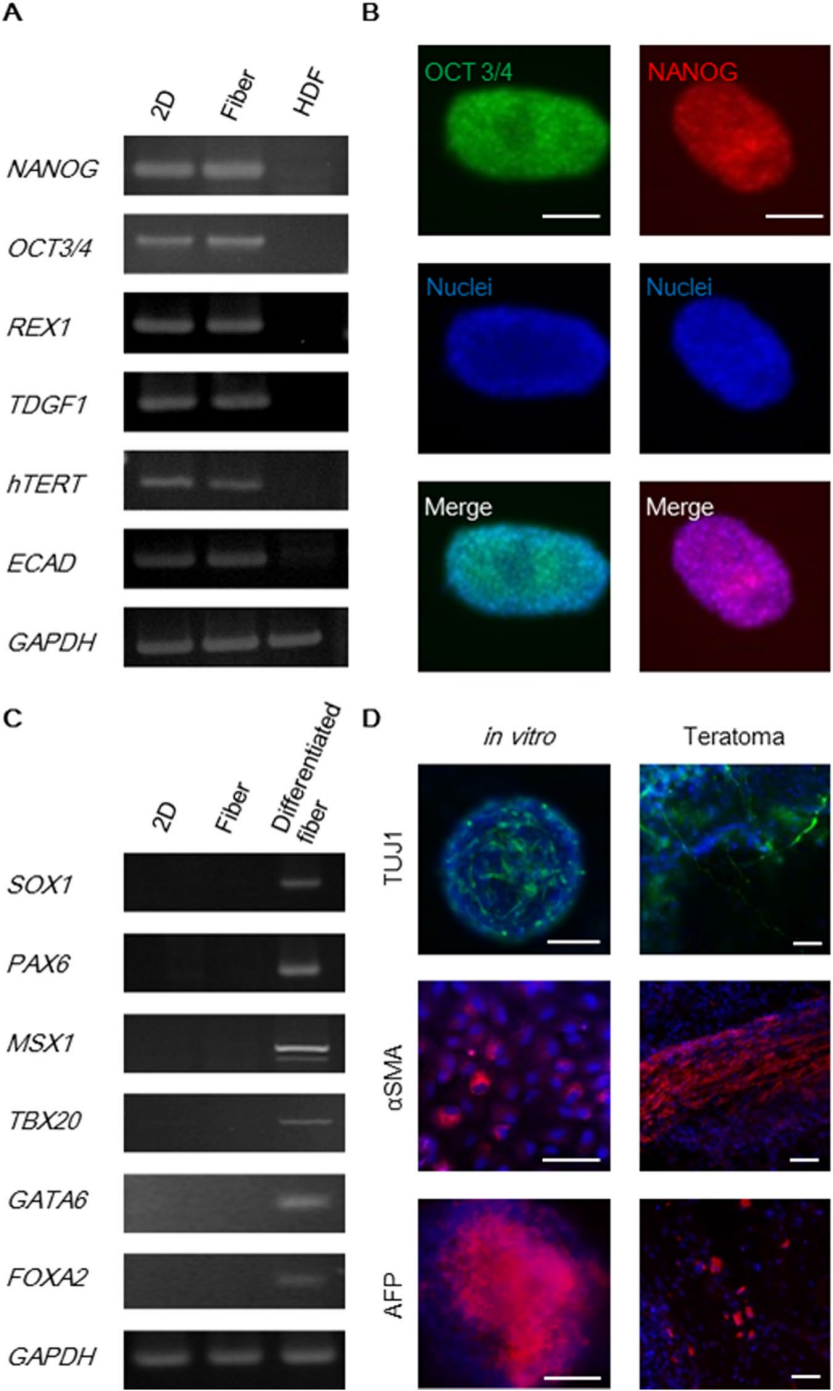
Characterization of the human iPS cells cultured in the core-shell microfiber culture system. (**A**) RT-PCR analysis of pluripotency-associated marker genes. 2D, cells cultured on a Matrigel-coated culture plate (positive control); Fiber, cells after culture for 44 days (11 passages) in the core-shell microfiber culture system; and HDF, human dermal fibroblasts (negative control). Raw data of gels are presented in Supplementary Figure S10. (**B**) Fluorescence images of cell aggregations stained for OCT3/4 (green) or NANOG (red) after culture for 44 days (11 passages). Nuclei were stained with Hoechst 33342 (blue). Scale bar: 100 μm. (**C**) RT-PCR analysis of differentiation marker genes. 2D, cells cultured on a Matrigel-coated culture plate (positive control); Fiber, cells after culture for 44 days (11 passages) in the core-shell microfiber culture system; and Differentiated fiber, cells cultured in the core-shell microfiber culture system with differentiation-induction medium after maintaining the culture for 48 days (12 passages) in the culture system. Full-length gels are presented in Supplementary Figure S11. (**D**) Fluorescence images of the *in vitro* differentiated fibers and teratoma using cells cultured for more than 32 days (8 passages) in the core-shell microfiber culture. The cells and tissues were stained with an ectoderm marker (TUJ1: green), a mesoderm marker (αSMA: red), an endoderm marker (AFP: red), and Hoechst 33342 (nuclei: blue). Scale bar: 100 μm.

To investigate whether human iPS cells sustained the properties of pluripotent stem cells after serial passaging in the core-shell microfiber culture system, we evaluated the expression of pluripotency-associated markers and the potency of the cells cultured through at least up to 32 says (8 passages). The expression levels of pluripotency-associated marker genes (*NANOG*, *OCT3/4*, *REX1*, *TDGF1*, *hTERT*, and *ECAD*) were high in the cells cultured in microfibers for 44 days (11 passages); these gene expression levels were similar to those in the cells cultured on a 2D surface (Fig. 6A). We also found that the cells expressed OCT3/4 and NANOG proteins, as detected by immunocytochemistry (Fig. 6B), and approximately 90% of the cells were positive for OCT3/4, SSEA-4, and TRA-1-60, as detected by flow cytometry (Supplementary Fig. S5). Moreover, our culture system retained the normal karyotype of the cells (Supplementary Fig. S6).

To confirm whether the cells retained pluripotency after serial passaging, we performed *in vitro* differentiation in the microfiber culture system and teratoma formation *in vivo*. After *in vitro* differentiation, we found that the cells expressed ectodermal (*SOX1* and *PAX6*), mesodermal (*MSX1* and *TBX20*), and endodermal (*GATA6* and *FOXA2*) marker genes (Fig. 6C). Furthermore, we found that the differentiated cells in both the microfiber and teratoma expressed ectodermal (TUJ1), mesodermal (αSMA), and endodermal (AFP) markers (Fig. 6D). These results demonstrated that the cells were able to sustain their properties for more than one month in the core-shell microfiber culture system.

## Conclusions

We have developed a core-shell microfiber culture system for highly efficient expansion of human iPS cells. The microfibers maintained the thickness of the cell aggregations in the core region, thereby improving the cell viability compared with the conventional suspension culture systems. Notably, human iPS cells accompanied with type I collagen at a low initial cell density demonstrated highly efficient expansion (14-fold in four days) while maintaining pluripotency even after serial passaging. These results suggest that our culture system may overcome the drawbacks of suspension culture systems such as cell death, low expansion rate, spontaneous differentiation, and abnormal karyotype of pluripotent stem cells after long-term culture^4, 8, 14, 15^. Therefore, the core-shell microfiber culture system is a feasible and highly efficient large-scale culture system for human pluripotent stem cells that can be used in regenerative medicine.

## Methods

### Cells

The human iPS cell line 409B2^31^ was obtained from RIKEN BioResource Center (Tsukuba, Japan). The cells were maintained on a Matrigel^®^ (BD Biosciences, Franklin Lakes, NJ)-coated 6-well plate or culture dish in the TeSR E8 medium (STEMCELL Technologies, Vancouver, Canada) under 5% CO_2_ at 37 °C and passaged every 4 days. For passaging on a two-dimensional (2D) surface, the cells were washed once in phosphate-buffered saline [PBS (−)] (Sigma-Aldrich, St. Louis, MO), incubated with the Accutase cell detachment solution (StemPro^™^ Accutase^®^, Thermo Fisher Scientific, Waltham, MA) for 5 min at 37 °C, and were then dissociated into single cells by pipetting. Detached cells suspended in the TeSR E8 medium were centrifuged at 400 × *g* for 3 min. The cells were seeded into a Matrigel-coated 6-well plate or culture dish and cultured in the TeSR E8 medium. ROCK inhibitor Y-27632 (Wako Pure Chemical Industries, Osaka, Japan) was added to the culture medium at 10 μM for the first 24 h in the culture to prevent dissociation-induced apoptosis^32^. The medium was replaced daily. For the suspension culture, cells were suspended in the TeSR E8 medium supplemented with 10 μM ROCK inhibitor and were cultured in low-attachment plates. Fifty percent of the medium was replaced daily starting from day 2 without adding ROCK inhibitor. For single-cell dissociation, the cell aggregations were incubated with Accutase for 5 min at 37 °C and then dissociated by pipetting. Human dermal fibroblasts (HDFs; Lonza, Basel, Switzerland) were grown on culture dishes in Dulbecco’s modified Eagle’s medium (DMEM; D6429, Sigma-Aldrich) containing 10% fetal bovine serum (HyClone™, GE Healthcare, Little Chalfont, UK) under 5% CO_2_ at 37 °C. HDFs were passaged every 7 days.

### Formation of core-shell hydrogel microfibers

The double-coaxial laminar-flow microfluidic device was fabricated by assembling pulled glass capillary tubes, rectangular glass tubes, and custom-made three-way connectors, as previously described^16^. For core-shell hydrogel microfiber formation, three solutions were prepared: (1) core solution: a cell suspension (1.0 × 10^7^ or 1.0 × 10^8^ cells/mL) for the core stream, (2) shell solution: a pre-gel solution of 1.5% Na-alginate (Wako Pure Chemical Industries) in saline for the shell stream, and (3) sheath solution: a mixture of 100 mM CaCl_2_ (Kanto Chemical, Tokyo, Japan) and 3% sucrose (Nacalai Tesque, Kyoto, Japan) solution for the sheath stream. Three different compositions of the core solution were used: (1) without ECM core solution, TeSR E8 medium containing cells; (2) Matrigel core solution, Matrigel solution containing cells; and (3) collagen core solution, 1 mg/mL neutralized type I collagen (AteloCell^®^, IPC-50, KOKEN, Tokyo, Japan) in the TeSR E8 medium containing cells. The flow rates of the core, shell, and sheath streams were 25 μL/min, 75 μL/min, and 3.6 mL/min, respectively. For the fabrication of human iPS cell-laden core-shell microfibers, the cells were pre-treated with 10 μM ROCK inhibitor for 1 h and then detached from the 2D surface. The single-cell suspension for the core solution was obtained by dissociation with Accutase cell detachment solution and was suspended in the TeSR E8 medium, Matrigel, or collagen. After encapsulation of the cell suspension, all core-shell microfibers were transferred to culture dishes and cultured in the TeSR E8 medium supplemented with 10 μM ROCK inhibitor. Fifty percent of the medium was replaced daily starting from day 2 without adding ROCK inhibitor. To form the microfibers containing fixed number of cells for evaluation of expansion rates, we injected the core solution with a fixed amount of cell suspension into the microfluidic device using pipettes before the microfiber fabrication. By using this method, approximately 96% of the injected cells were successfully retrieved from the microfibers immediately after fabrication. The data indicated that the cell loss through encapsulation and retrieval process was less than 4% (Supplementary Fig. S7). We used 4 mL of the TeSR E8 medium to initiate culturing of the microfibers containing 5.0 × 10^5^ cells.

### Retrieval of cells from the core-shell microfibers and serial passaging

For the removal of the calcium alginate shell, 4 mg/mL alginate lyase (Sigma-Aldrich) in PBS (−) was added at a 1:100 ratio to the culture medium containing the core-shell microfibers. The microfibers were then incubated at 37 °C for 5 min to enzymatically digest the outer alginate shell. The human iPS cell aggregations retrieved from the microfibers were dissociated into single cells by treatment with the Accutase cell detachment solution for 5 min (Supplementary Fig. S8). Viable cells were counted by a trypan blue (Life Technologies, Carlsbad, CA) exclusion assay. For serial passaging, the cell-laden core-shell microfibers were pre-treated with 10 μM ROCK inhibitor for 1 h before single-cell dissociation. The single cells were then suspended in 1 mg/mL collagen solution and re-encapsulated within the microfibers in the same way as shown in formation of core-shell hydrogel microfibers.

### Viability assay for human iPS cell fibers

Viability assay was performed on human iPS cells in the suspension culture system and the core-shell microfiber culture system using a Live/Dead Viability/Cytotoxicity kit (Life Technologies). For quantitative evaluation of cell viability, we stained the single-cell suspension obtained from the cell-laden microfibers and the cell aggregations in the suspension culture. To prepare the single-cell suspension, the aggregations were dissociated by treatment with the Accutase cell detachment solution for 5 min. Cell aggregations and single-cell suspensions were incubated with the live/dead viability assay working solution for 15 min at 37 °C. The cells were imaged and counted by phase-contrast and fluorescence microscopy.

### RT-PCR

Total RNA was isolated by the Trizol^®^ reagent (Life Technologies) and was then treated with recombinant DNase I (Takara Bio, Otsu, Japan) to eliminate genomic DNA contamination. Five hundred nanograms of total RNA was used for the reverse transcription reaction with PrimeScript™ reverse transcriptase (Takara Bio), according to the manufacturer’s instructions. PCR was performed with Ex Taq^®^ DNA polymerase (Takara). Primer sequences, annealing temperatures, and PCR cycle numbers are provided in Supplementary Table S1.

### *In vitro* differentiation

Human iPS cell-laden core-shell microfibers accompanied with type I collagen were cultured in the TeSR E8 medium for four days. Subsequently, the microfibers were transferred to another dish and cultured in a differentiation-induction medium (DMEM containing 20% fetal bovine serum) for another 16 days. The medium was replaced every three days. After culture, cell aggregations were retrieved by digesting the alginate shell, transferred to an iMatrix-511 (Nippi, Tokyo, Japan)-coated plate, and cultured overnight in the same medium under 5% CO_2_ at 37 °C to allow cell adhesion for immunocytochemistry. For embryoid body (EB) differentiation, we used the cell aggregations formed by the hanging drop method and cultured the cell aggregates in the differentiation-induction medium for another 16 days.

### Teratoma formation

For teratoma formation assay, human iPS cells that dissociated into single cells after long-term culture in our culture system were injected directly into the subrenal capsular space of immunodeficient mice (C.B-17/lcr-scid/scidJcl; CLEA Japan, Tokyo, Japan). After 3 months, the teratomas were surgically dissected out of the mice and fixed with 4% paraformaldehyde solution (Wako Pure Chemical Industries). For immunocytochemistry, each fixed teratoma was sectioned into 7-μm slices using a cryostat. All animal experiments in this study were approved in advance by the University of Tokyo and were conducted in accordance with rules of its Institutional Animal Care and Use Committee.

### Immunostaining

Samples were washed with PBS, fixed with 4% paraformaldehyde solution (Wako Pure Chemical Industries) for 20 min, permeabilized with 1% Triton™ X-100 (Sigma-Aldrich) in PBS for 20 min, and then stained overnight with the following antibodies: phycoerythrin (PE) mouse anti-human NANOG, Alexa Fluor^®^ 488 mouse anti-OCT3/4 (BD Biosciences), mouse anti-TUJ1 (β-tubulin III; Sigma-Aldrich), rabbit anti-α-SMA (PA1-37024, Thermo Fisher Scientific), and rabbit anti-AFP (Abcam, Cambridge, UK). The secondary antibodies Alexa Fluor^®^ 488 goat anti-mouse IgG (Thermo Fisher Scientific) and Alexa Fluor^®^ 568 Goat Anti-Rabbit IgG (Thermo Fisher Scientific) were then added. The nuclei were stained with Hoechst 33342 (Lonza).

### Flow cytometry

Cells were dissociated with the Accutase cell detachment solution for 5 min. The dissociated human iPS cells were washed with PBS, fixed with 4% paraformaldehyde solution for 20 min, permeabilized with permeabilization/wash buffer (BD Biosciences) for 20 min, and then stained with PE mouse anti-SSEA-4, FITC mouse anti-human TRA-1-60, and Alexa Fluor^®^ 488 mouse anti-OCT3/4 (BD Biosciences) for 1 h on ice. Stained cells were then analysed using the FACSVerse™ and FACSuite™ software.

## Electronic supplementary material

Supplementary Information
