# Supplementary material for: Cell fiber-based three-dimensional culture system for highly efficient expansion of human induced pluripotent stem cells
Source: Sci Rep. 2017 Jun 6;7:2850. doi: 10.1038/s41598-017-03246-2 (PMC5460280; doi:10.1038/s41598-017-03246-2)
Supplement: Supplementary file 1 — Supplementary Information [file 41598_2017_3246_MOESM1_ESM.pdf]

Supplementary information

Title

Cell fiber-based three-dimensional culture system for highly efficient expansion of human induced pluripotent stem cells

Authors

Kazuhiro Ikeda[1, 3], Shogo Nagata[1, 2], Teru Okitsu[1, 2], Shoji Takeuchi[1, 2]*

[1]Institute of Industrial Science, The University of Tokyo, Tokyo, Japan

[2]ERATO Takeuchi Biohybrid Innovation Project, Japan Science and Technology Agency, Tokyo, Japan

[3]Graduate School of Life and Environmental Sciences, University of Tsukuba, Ibaraki, Japan

* e-mail: takeuchi@iis.u-tokyo.ac.jp; Tel.: +81-3-5452-6650; Fax: +81-3-5452-6649

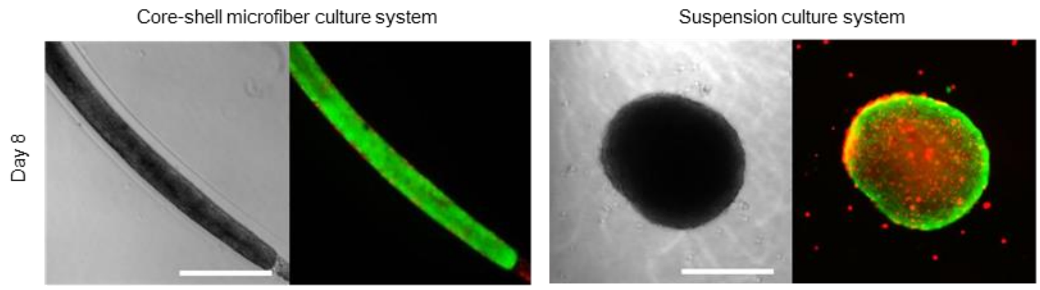

Supplementary Figure S1. Live/dead staining of human induced pluripotent stem (iPS) cells cultured in the core-shell microfiber culture system using a core-shell microfiber without additional extracellular matrix (ECM) components and conventional suspension culture system on day 8. Live cells were stained with calcein AM (green) and dead cells were stained with ethidium homodimer (red). Scale bar: 500 μm.

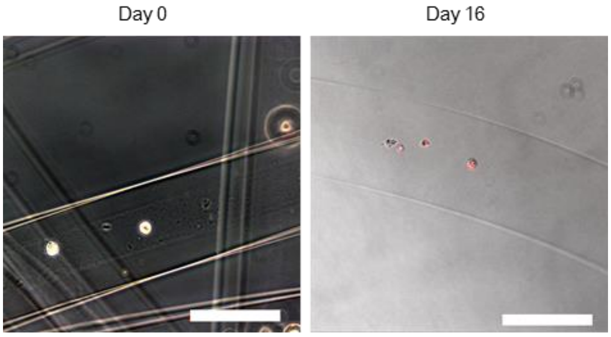

Supplementary Figure S2. Typical phase-contrast image (day 0) and merged phase-contrast and fluorescence image (day 16) of human iPS cells cultured in the microfibers at very low initial cell density (1.0×10^6 cells/mL). The cells on day 16 were stained by ethidium homodimer (red), indicating non-viable cells. Scale bar: 200 μm.

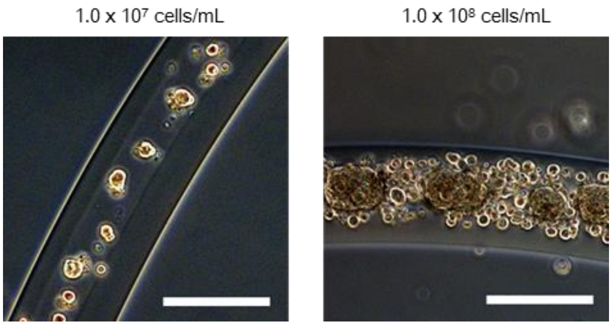

Supplementary Figure S3. Human iPS cells accompanied with collagen at low (1.0 × 10^7 cells/mL) and high (1.0 × 10^8 cells/mL) initial cell density on day 1. Scale bar: 250 μm.

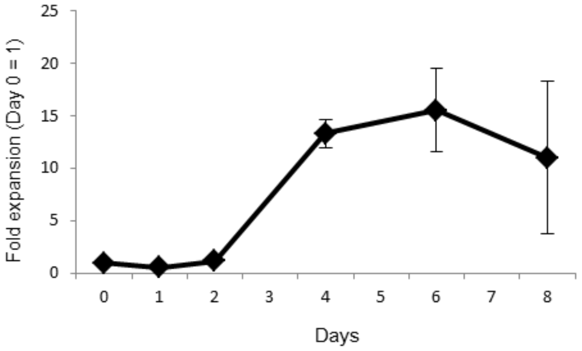

Supplementary Figure S4. Growth kinetics of human iPS cells in the core-shell microfiber culture system until day 8 (N ≥ 3).

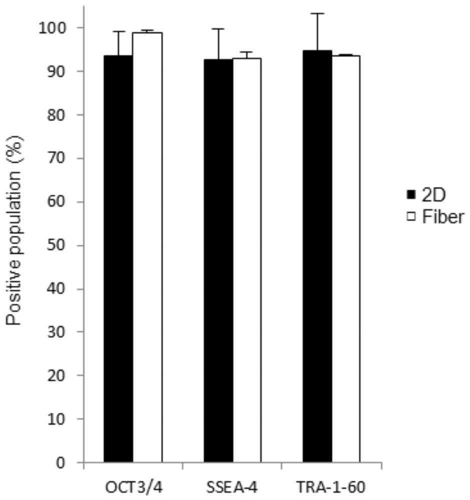

Supplementary Figure S5. Flow cytometry analysis of pluripotency-associated markers (OCT3/4, SSEA-4, and TRA-1-60). Cells cultured in the conventional 2D culture system (2D) or in the core-shell microfiber culture system (Fiber) for more than one month were analysed (N ≥ 3).

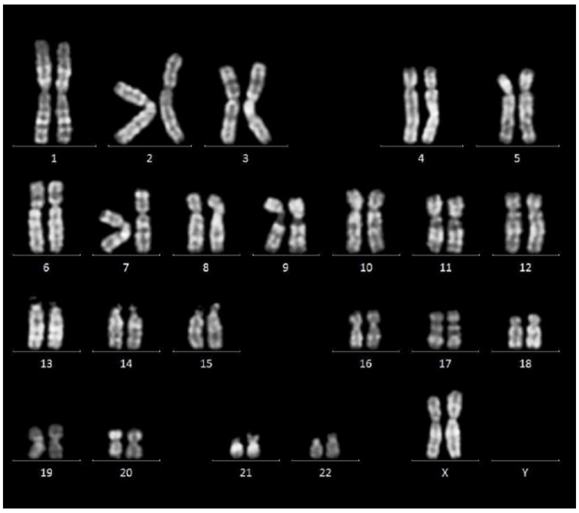

Supplementary Figure S6. Karyotyping of human iPS cells cultured for 56 days (14 passages) in the core-shell microfiber culture system by Q-band analysis (N = 20).

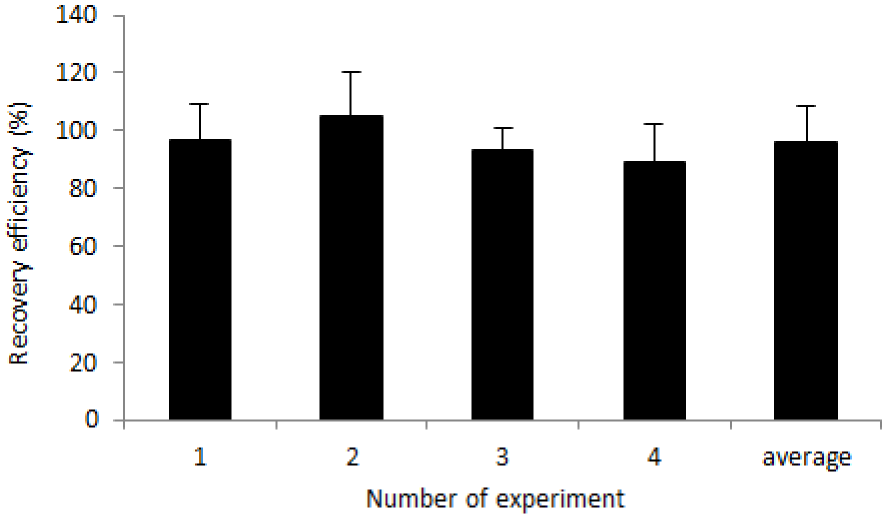

Supplementary Figure S7. Recovery efficiency of the encapsulated cells from the core-shell microfibers. To release the cells from the microfibers, the alginate shell was dissolved using alginate lyase (Sigma-Aldrich), as previously described[16]. The values were obtained from retrieved viable cell number immediately after the microfiber fabrication and estimated encapsulated cell number (N = 3).

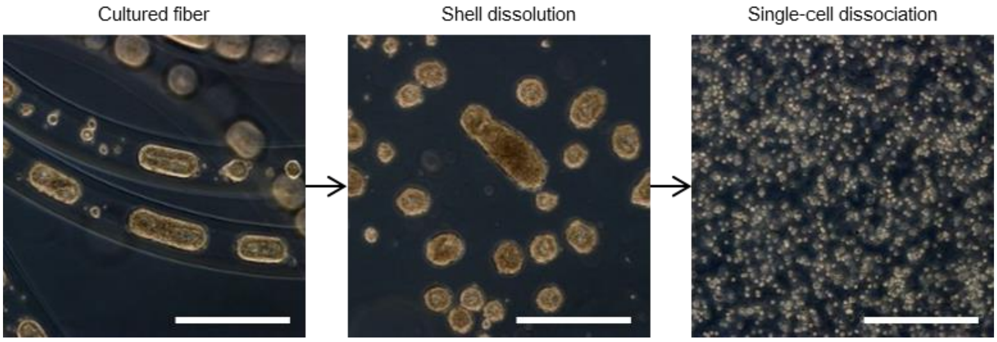

Supplementary Figure S8. Human iPS cells in the microfibers were retrieved by shell dissolution using alginate lyase and dissociated into single cells using the Accutase cell detachment solution. Scale bar: 500 μm.

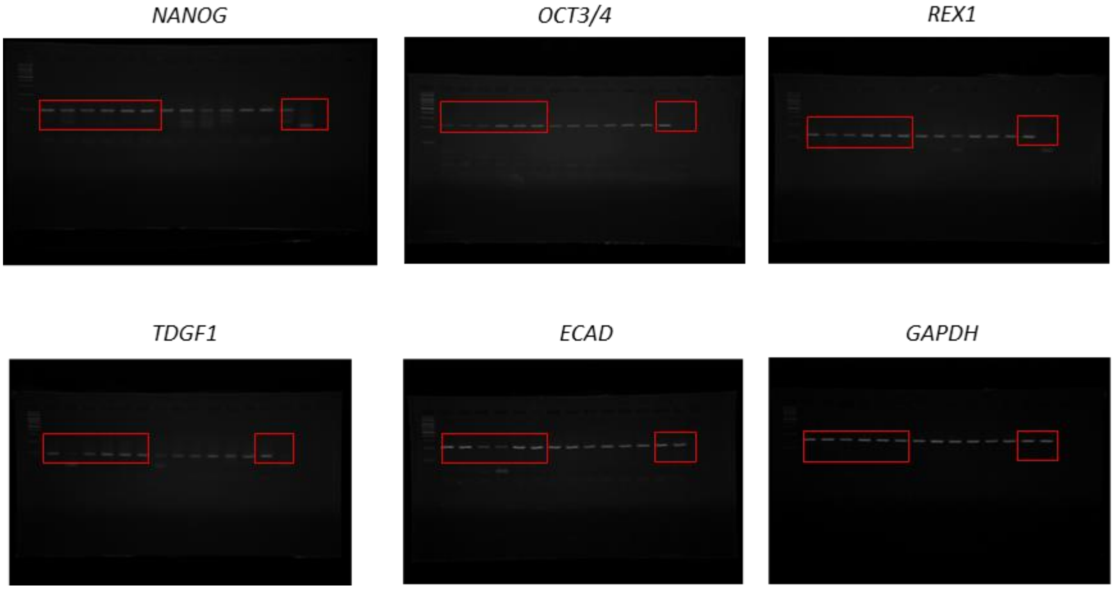

Supplementary Figure S9. Full images of gels in Fig. 4B.

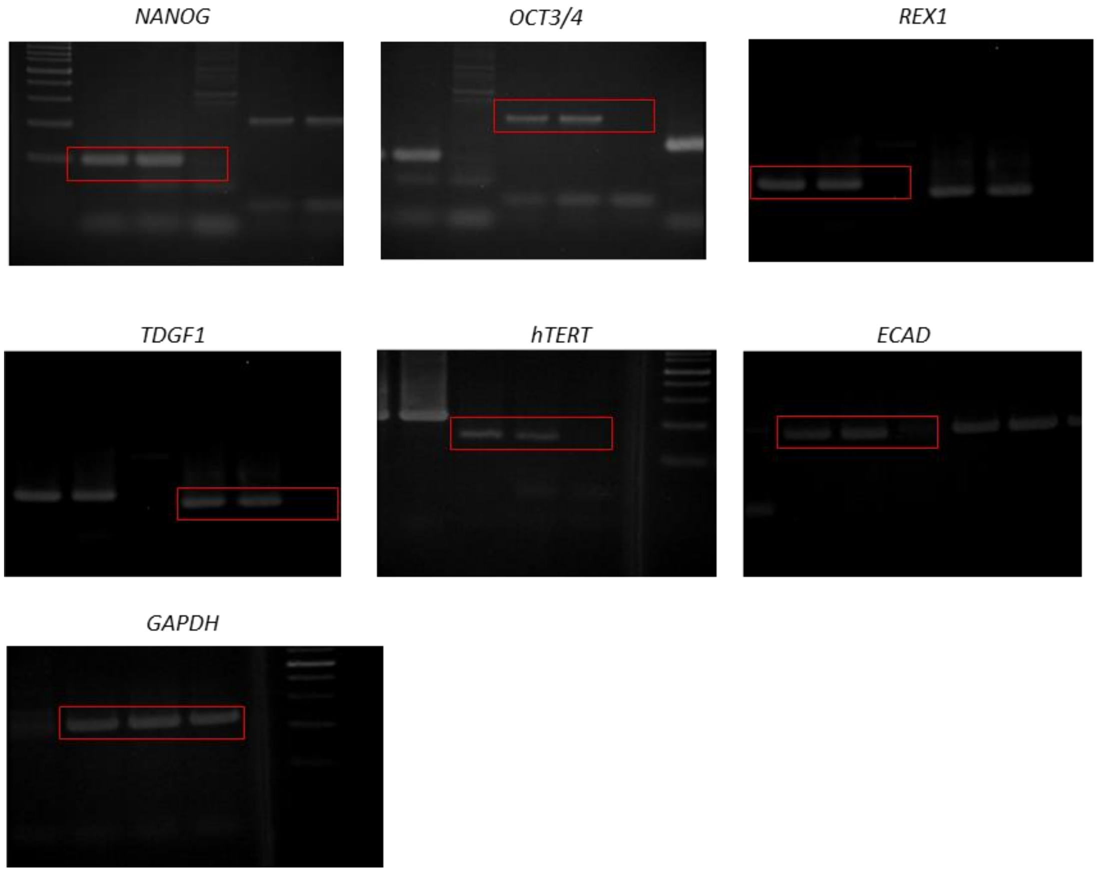

Supplementary Figure S10. Raw data of gels in Fig. 6A.

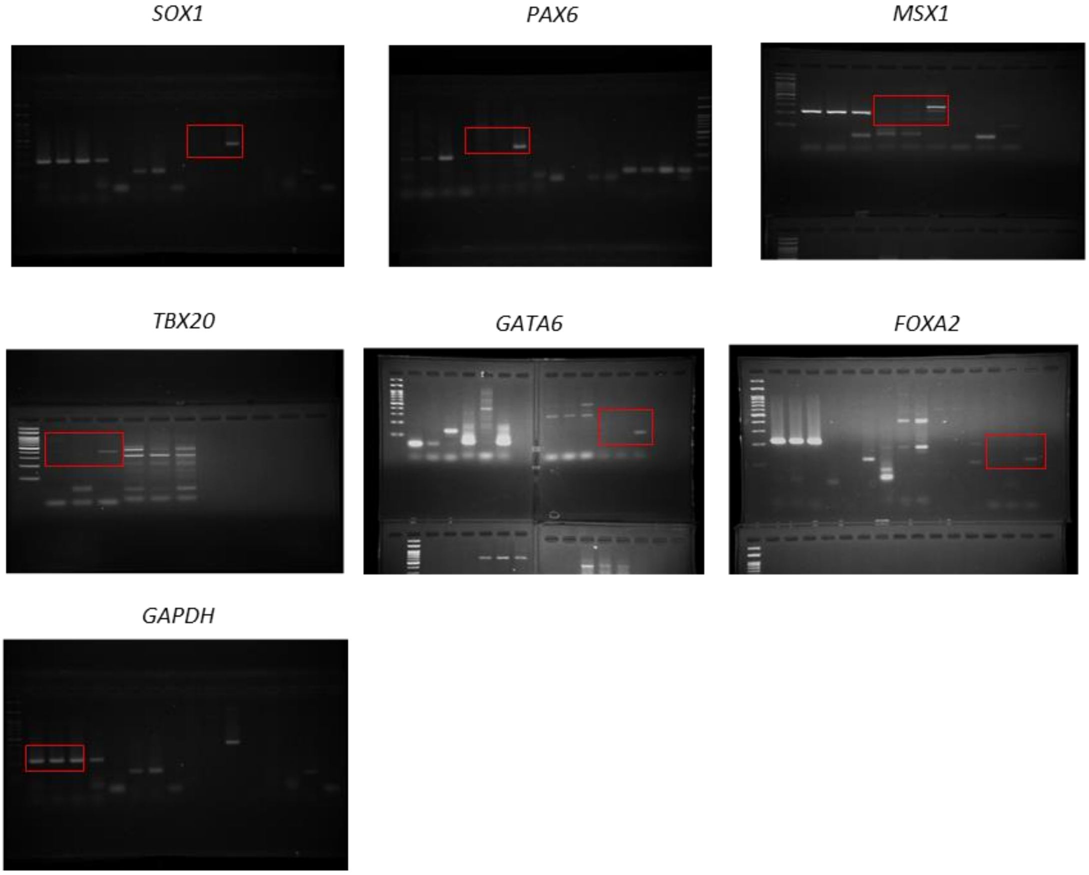

Supplementary Figure S11. Full images of gels in Fig. 6C.

Genes	Forward primer 5' - 3'	Reverse primer 5' - 3'	Annealing (℃)	cycle
NANOG	CTGCTGAGATGCCTCACACG	TGCCTTTGGGACTGGTGGA	55	30
OCT3/4	TCTCGCCCCCTCCAGGT	GCCCCACTCCAACCTGG	55	30
REX1	AGAAACGGGCAAAGACAAGAC	GCTGACAGGTTCTATTTCCGC	55	30
TDGF1	ACAGCACAGTAAGGAGCTAAAC	CGTCCGTAGAAGGAGGGAGG	55	30
ECAD	GAGCTTGTCATTGAGCCTGGCA	TGGGCAAATGTGTTCAGCTCAGC	55	30
hTERT	TGTGCACCAACATCTACAAG	GCGTTCTTGGCTTTCAGGAT	55	30
SOX1	GCGGAAAGCGTTTTCTTTG	TAATCTGACTTCTCCTCCC	55	30
PAX6	ACCCATTATCCAGATGTGTTTGCCCGAG	ATGGTGAAGCTGGGCATAGGCGGCAG	55	30
MSX1	CGAGAGGACCCCGTGGATGCAGAG	GGCGGCCATCTTCAGCTTCTCCAG	60	35
TBX20	AGGTACCGCTACGCCTAC	GTCAGTGAGCCTGGAGGA	60	35
GATA6	AGGGCTCGGTGAGTCCAAT	CGCTGCTGGTGAATAAAAAGGA	55	30
FOXA2	TGGGAGCGGTGAAGATGGAAGGGCAC	TCATGCCAGCGCCCACGTACGACGAC	60	30
GAPDH	CTTCTTTTGCGTCGCCAGCCGAG	CAGCCTTGACGGTGCCATGGAA	55	30

Supplementary Table S1. List of primers used for semi-quantitative reverse RT-PCR.
